# Flowering Periods, Seed Yield Components, Seed Quality, and Patterns of Seed Shattering in *Paspalum*: Effect of Taxonomy and Nitrogen Fertilization

**DOI:** 10.3390/plants13172411

**Published:** 2024-08-29

**Authors:** Luis Leandro Chamorro, Elsa Andrea Brugnoli, Alex Leonel Zilli, Roberto Ramón Schulz, Florencia Marcón, Carlos Alberto Acuña

**Affiliations:** Instituto de Botánica del Nordeste, Consejo Nacional de Investigaciones Científicas y Técnicas, Facultad de Ciencias Agrarias, Universidad Nacional del Nordeste, Sargento Cabral 2131, Corrientes P.C. 3400, Argentina; luis90chamorro@gmail.com (L.L.C.); abrugnoli@agr.unne.edu.ar (E.A.B.); azilli@agr.unne.edu.ar (A.L.Z.); roberto@cardinalagrogestion.com (R.R.S.); fmarcon91@gmail.com (F.M.)

**Keywords:** *Paspalum notatum*, Plicatula group, density of inflorescences, seed retention

## Abstract

Perennial warm-season grasses typically have reduced seed yield, making it essential to identify the critical seed yield components. An induced increase in nitrogen could help determine which components are most limiting. This research aimed to estimate seed yield components in *Paspalum*; evaluate N fertilization effects on the reproductive phase, seed yield components, and seed quality; and establish the pattern of seed shattering over time. Nine genotypes covering different reproductive periods were used. The experimental design was a randomized complete block design in a split-plot arrangement with three replications. The main plots had two nitrogen levels (0 and 150 Kg N ha^−1^), and the sub-plots contained different genotypes. Seed yield variation was mainly related to reproductive tiller density among germplasm with different flowering periods. Early-flowering germplasm showed an extended flowering period (159%), greater tiller density (27.7%), greater reproductive tiller density (157%), and higher yield (302%) in response to nitrogen fertilization. Seed-quality traits and seed retention were not affected by nitrogen fertilization. Seed retention over time followed an inverted sigmoid pattern, though there was considerable variation among taxonomic groups. Early-flowering germplasm exhibited superior seed retention. Seed yield in *Paspalum* is mainly influenced by the density of reproductive tillers and seed retention.

## 1. Introduction

The warm-season (C_4_) grasses are the major forage resources for ruminant livestock in the tropical and subtropical world. In addition, many of these species are also used for turf in warm and temperate regions [1]. Most perennial warm-season grasses have not been domesticated, and they still carry many undesirable traits that affect seed yield and quality. For example, although the genus *Urochloa* includes some of the most important forages worldwide, the seed for this group of species is mostly recovered from the ground [2]. An extended reproductive phase and low seed retention are common traits not only in *Urochloa* but also in the most important forage and turf species. Low seed fertility is also common because warm-season grasses are mainly polyploid, and meiotic irregularities are characteristic [3]. Moreover, polyploidy usually disrupts the normal process of meiosis by complicating chromosome pairing and segregation, leading to meiotic irregularities, such as multivalent formation, non-disjunction, and the production of unbalanced gametes [4]. Apomixis is well represented among these species, and it usually helps to overcome seed sterility. Dormancy is also present in different forms among warm-season grasses, as a survival mechanism that has made them successful in nature, but results in a slow establishment when they are cultivated [5].

The genus *Paspalum* (Poaceae) represents an interesting model to study the main ecological and genetic factors involved in seed yield and seed quality in warm-season grasses. The genus has nearly 330 species that have different flowering periods when grown in the subtropics. *Paspalum* has a vast distribution across the Americas, and many of its species play key ecological roles in extensive grasslands and rangelands. Several *Paspalum* species are cultivated as cereal, forage, and turf worldwide [6]. *Paspalum notatum* has been characterized as a long-day species, while *P. atratum* as responsive to a combination of long and short days [7]. Moreover, there are species, such as *P. guenoarum,* that flower between *P. notatum* and *P. atratum,* and it seems to be a medium-day species [8]. *P. notatum*, *P. guenoarum,* and *P. atratum* are tetraploid and apomictic as are many warm-season perennial grasses. Intra- and inter-specific hybrids have been generated for these species with the objective of developing new forage cultivars [9,10]. This diverse germplasm should be useful to determine if there are common ecological factors affecting seed-related traits in *Paspalum*. This information is expected to allow for a better definition of breeding objectives, aiming to improve seed yield in warm-season perennial grasses.

Crop yield increase results from improving resource availability, mainly water and nitrogen, combined with the adoption of more responsive cultivars [11]. These increases in grain or seed yields are the result of changes in yield components. Slafer et al. [12] analyzed a large set of data and stated that wheat yield is mainly regulated by changes in grains per m^2^ and spikes per m^2^. Perennial C_4_ species seem to respond similarly. An increase in inflorescence density was related to greater seed yields in *Paspalum notatum* [13,14]. However, floral initiation in the available vegetative tillers is expected to respond differently between annual and perennial grasses. Greater nitrogen availability resulted in higher tiller density and reproductive differentiation within the available tillers in *P. notatum* [15]. Considering that *Paspalum* has a large number of species with different flowering periods, it would be of interest to evaluate if there is a common response among them. A high amount of N can be applied to low-fertility soil to induce a change in seed yield, which may help determine if there are common critical seed yield components among *Paspalum* species.

Seed shattering is the physical separation of the seed from the parental plant [4]. In the C_4_ species, seed separation results from disarticulation of the rachis just below the glumes. Since most perennial warm-season grasses have an extended reproductive phase, high seed shattering results in low amounts of seed harvested [16]. Moreover, the forage seed industry in tropical South America is mainly based on equipment developed for recovering the seed from the soil’s surface with all the environmental implications related to this practice [17]. Tomás et al. [18] found diversity for seed retention (the opposite of seed shattering) within the germplasm of *Panicum coloratum*. Although marked differences have been reported for seed yield among *Paspalum* species [8,19], the impact of seed shattering and the potential variability among species and environments have not been documented. An interesting method for evaluating seed shattering was developed by Tomás et al. [18] for *Panicum coloratum,* which can be adjusted to investigate this trait in *Paspalum* species.

The objectives of this research were to (1) determine the flowering periods of different *Paspalum* genotypes, (2) estimate the seed yield components responsible for changes in seed yield, (3) evaluate the effect of nitrogen fertilization on the extent of the reproductive phase, seed yield components, and seed quality, and (4) establish the pattern of seed shattering over time.

## 2. Results

### 2.1. Flowering Period

**Figure 1:**
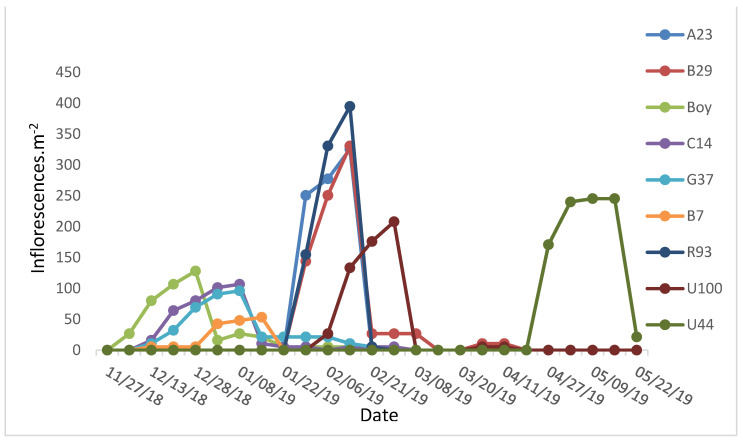
Number of inflorescences per m^2^ for 9 genotypes of *Paspalum* during the 2018–2019 growing season in Subtropical Argentina. Boy, C14, G37, and B7 belong to *P. notatum* (early flowering), and R93, U100, U44, A23, and B29 belong to the Plicatula Group (intermediate and late flowering).

Three flowering periods were observed among the *Paspalum* genotypes included in this research (Figure 1). The *Paspalum notatum* hybrids (early-flowering group) started flowering in late spring and lasted until the end of January, with a maximum at the end of December. The second group (intermediate-flowering group) included a *P. guenoarum* ecotype and *P. plicatulum* × *P. guenoarum* hybrids. Inflorescences for this second group were registered between late January and early March. However, a low number of inflorescences kept emerging for U100 and R93 until mid-April and mid-May, respectively. The maximum number of inflorescences was observed during February. The third group (late-flowering group) was represented by an ecotype of *P. atratum*. The reproductive phase for this group was registered between mid-April and mid-May. The maximum density of inflorescences was greater for the intermediate- and late-flowering groups in comparison with the early flowering-group (Figure 1). No significant differences were observed for the extent of the reproductive phase between genotypes or groups.

**Figure 2:**
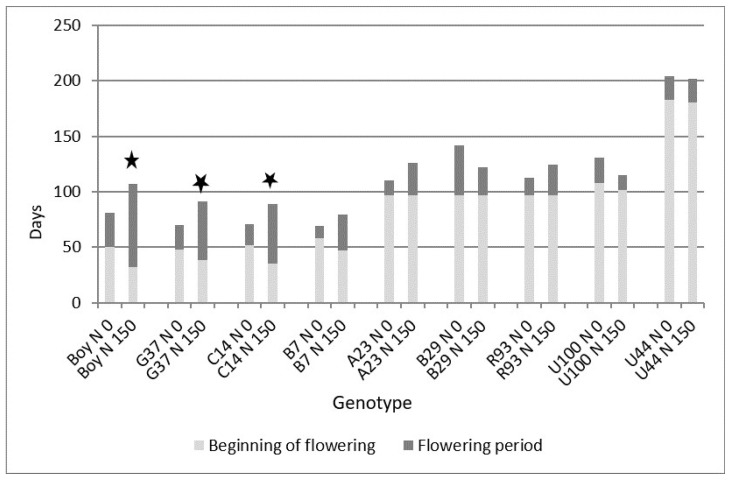
Effect of nitrogen fertilization (N 0–N 150: 0 and 150 Kg N ha^−1^) on the time to beginning and extent of flowering in a group of 9 *Paspalum* genotypes. Boy, C14, G37, and B7 belong to *P. notatum* (early flowering), and R93, U100, U44, A23, and B29 belong to the Plicatula Group (intermediate and late flowering). The stars indicate significant differences in the extent of the flowering period between nitrogen levels for each genotype (Tukey test (*p* ≤ 0.05)).

Nitrogen fertilization resulted in an earlier initiation of flowering in the *P. notatum* hybrids (Figure 2). The inflorescences emerged between 10 and 18 d before the control. Moreover, the flowering period lasted between 21 and 45 more days in comparison with the control without N fertilizer. No significant effect of N fertilization was observed for the initiation and extension of the flowering period for the intermediate- and late-flowering groups (Figure 2).

### 2.2. Seed Yield and Yield Components

Nitrogen had an impact on seed yield mainly in the early-flowering group, increasing seed yield between 3 and 6.7 fold (Table 1). Significant differences between N-fertilized and non-fertilized plots were observed for Boyero, C14, and B7. Nitrogen did not have a significant effect on seed yield for genotypes of the intermediate- and late-flowering groups. Marked differences in favor of the intermediate- and late-flowering groups were observed for seed yield in comparison to the early-flowering group within non-fertilized plots. However, smaller differences were observed among groups in fertilized plots (Table 1). Variability was also observed within the early-flowering group in fertilized plots.

Nitrogen fertilization did not have a significant effect on the number of racemes per tiller, number of seeds per inflorescence, seed weight, number of vegetative tillers, and seed set for any of the evaluated genotypes. The number of reproductive tillers was significantly affected by nitrogen in four genotypes. Two belong to the early-flowering group (Boy and C14, with increases from 192 to 571 and from 160 to 464, respectively) and two to the intermediate-flowering group (A23 and R93, with increases from 325 to 890 and from 347 to 736, respectively). Although significance was detected only for two genotypes of the early-flowering group, the number of reproductive tillers increased between 2 and 3 fold. The same genotypes (Boyero and C14) that exhibited significant differences in seed yield also showed differences in tiller density (Table 1). In contrast, the two genotypes belonging to the intermediate-flowering group (A23 and R93) that exhibited differences in reproductive tiller density between the fertilized and non-fertilized plots did not show differences in seed yield.

### 2.3. Seed Dimensions and Quality

A significant effect of nitrogen fertilization for seed length was only detected for G37 and A23, but the increment represented 2.7% in one case and 5% in the other (Table 1). Nitrogen significantly increased seed width by 3.4% in the case of C14 and decreased by 5% in the case of U100 (Table 1).

Nitrogen fertilization did not have an effect on seed germination and the rate of germination (Table 1). Genotypes from the early-flowering group exhibited lower germination and rate of germination in comparison to genotypes from the intermediate- and late-flowering groups. Additionally, lower germination and rate of germination were observed for A23 (21.3 and 1.59) in comparison to U100 (47.3 and 4.13) and U44 (44 and 3.72, respectively) within the intermediate- and late-flowering groups.

### 2.4. Seed Shattering

**Figure 3:**
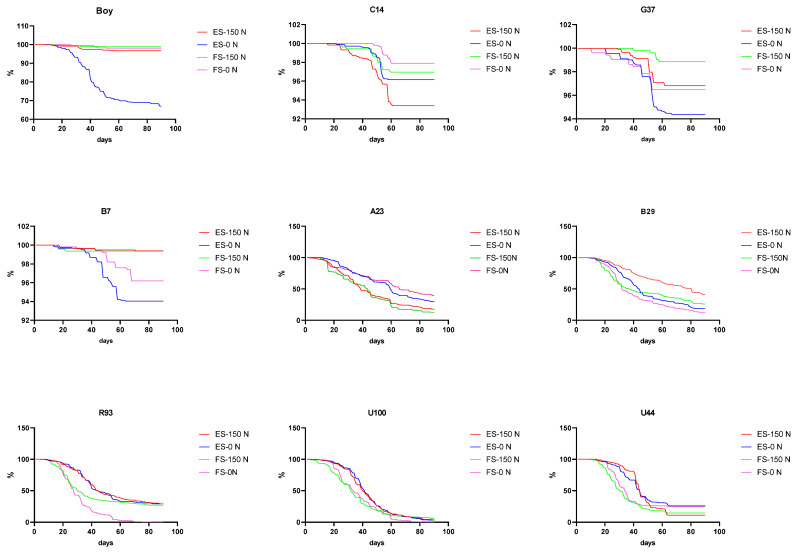
Seed shattering over time for 9 *Paspalum* genotypes cultivated on nitrogen-fertilized and non-fertilized plots. Boy, C14, G37, and B7 belong to *P. notatum* (early flowering), and R93, U100, U44, A23, and B29 belong to the Plicatula Group (intermediate and late flowering). ES-150N: empty spikelets fertilized with 150 kg N, ES-0N: empty spikelets non-fertilized, FS-150N: full seed fertilized with 150 kg N, FS-0N: full seed non-fertilized.

**Figure 4:**
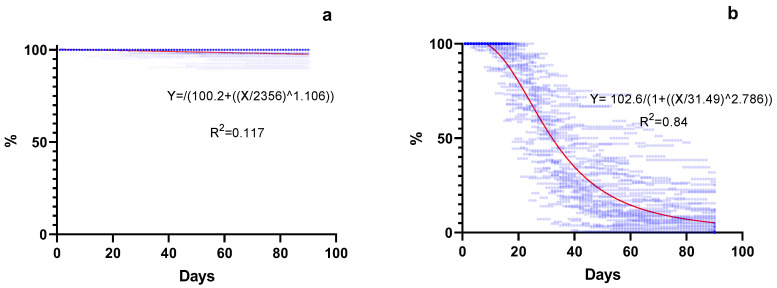
Seed retention over time in *Paspalum*. (**a**) Group of 4 genotypes of tetraploid *Paspalum notatum* (early flowering); (**b**) group of 5 tetraploid genotypes from the Plicatula Group (intermediate and late flowering).

Marked variation was observed for seed shattering 90 d post anthesis among the 9 evaluated genotypes, (from 1% to 100%) (Figure 3 and Table 2). Seed retention was greater for the early-flowering group (96–99%) compared to the intermediate- (1–19%) and late-flowering (0–1%) groups during the entire evaluation period (Table 2). Differences between groups were evident as early as 20 days after anthesis (Figure 3). At 60 d post anthesis, between 1.6 and 20 times more retention was observed for genotypes from the early-flowering group in comparison with the intermediate- and late-flowering groups. By 90 d after anthesis, the early-flowering group retained between 3.3 and 96 times more seed in comparison with genotypes from the intermediate- and late-flowering groups. Empty spikelets tend to shatter at a similar rate to full seeds (Figure 3). Seed-retention functions were developed for the early-flowering group and the intermediate- and late-flowering groups (Figure 4a,b, respectively).

Nitrogen fertilization did not significantly affect seed retention in most of the evaluated cases. There was an exception; B29 exhibited greater retention after 90 d when fertilized. The rate of seed retention was similar for both the fertilized and non-fertilized plots.

### 2.5. Relationship among Evaluated Variables

**Figure 5:**
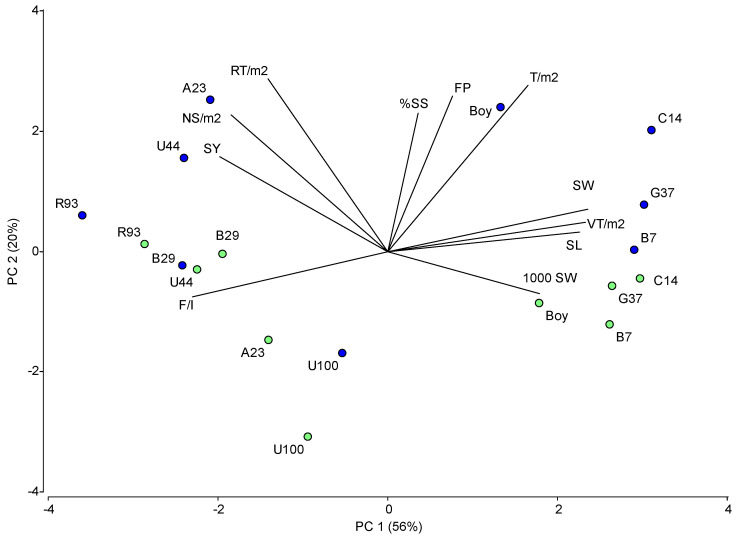
Principal component analysis (PCA) of 11 seed-related traits measured for 9 genotypes of *Paspalum* across two N-fertilization rates (0 and 150 Kg N ha^−1^). Boy, C14, G37, and B7 belong to *P. notatum* (early flowering), and R93, U100, U44, A23, and B29 belong to the Plicatula Group (intermediate and late flowering). Different colors represent different genotypes with different nitrogen levels, blue: 150 kg ha^−1^, green: 0 kg ha^−1^. Measured traits were %SS: percentage of seed set, FP: flowering period, F/I: N° flowers/inflorescence, T/m^2^: N° tillers per m^2^, RT/m^2^: reproductive tiller per m^2^, VT/m^2^: vegetative tillers per m^2^; SY: seed yield, NS/m^2^: N° of seeds per m^2^, SW: seed width, SL: seed length, 1000 SW: 1000 seed weight.

All seed-related traits contributed similarly to the observed variation (Figure 5). The number of seeds per m^2^ was the most closely related to seed yield, and the number of inflorescences per m^2^ explained most of this relationship. In contrast, the number of vegetative tillers was negatively related to seed yield. Seed weight, length, and width were also negatively related to seed yield (Figure 5).

Variability among groups was observed, with the early-flowering group distributed into the right two quadrants and the intermediate- and late-flowering groups into the left two quadrants (Figure 5). In addition, within the genotypes, more differences between the nitrogen-fertilized and non-nitrogen-fertilized groups were observed in early flowering genotypes. The intermediate- and late-flowering genotypes did not exhibit a clear positive impact of N-fertilization on the seed yield components, with the exception of A23 and U44.

## 3. Discussion

Although forage yield is the main focus for the genetic improvement of forage species, seed yield is essential for commercialization and good establishment of new cultivars [5,20]. In this research, we explored the main seed yield components affecting yield in *Paspalum* using a set of ecotypes and hybrids expected to cover the different reproductive periods across the growing season in the subtropics. Also analyzed was the effect of nitrogen fertilization on the extent of the reproductive phase and seed yield and quality. Lastly, the pattern of seed shattering for a period of 90 d post-anthesis was determined.

The germplasm used in this research represented early-, intermediate-, and late-flowering groups. This allowed for an evaluation of the responses across most of the growing season. Although the genotypes among groups did not differ in the extension of the reproductive phase, the density of inflorescences was greater for the intermediate- and late-flowering groups, particularly during the flowering peak, indicating greater seed yield potential. The addition of nitrogen to the system interacted with the photoperiod sensitivity of the early-flowering group (*P. notatum* hybrids), resulting in an earlier initiation of flowering, a later end of flowering, and a more extended flowering period. Since *P. notatum* behaves as a long-day species [5], these results indicate that the critical day length is lower with higher soil fertility. Nitrogen fertilization may help to induce flowering in *P. notatum* and facilitate seed production in more tropical zones. In contrast, an increase in the extent of the reproductive phase is undesirable for maintaining high forage nutritive value. Since the intermediate and late flowering groups were not responsive to the N treatment, it can be stated that only the extent of the reproductive phase of the early-flowering group is expected to be modified depending on nitrogen availability. The lack of response of the intermediate- and late-flowering groups to N fertilization may be related to their differential response to day length. It has been reported that *P. atratum* has a concentrated flowering period at the end of the growing season, since it responds to a combination of long and short days [7]. This differential response between *P. notatum* and *P. atratum* to day length may play a key role in the effect of N fertilization.

**Figure 6:**
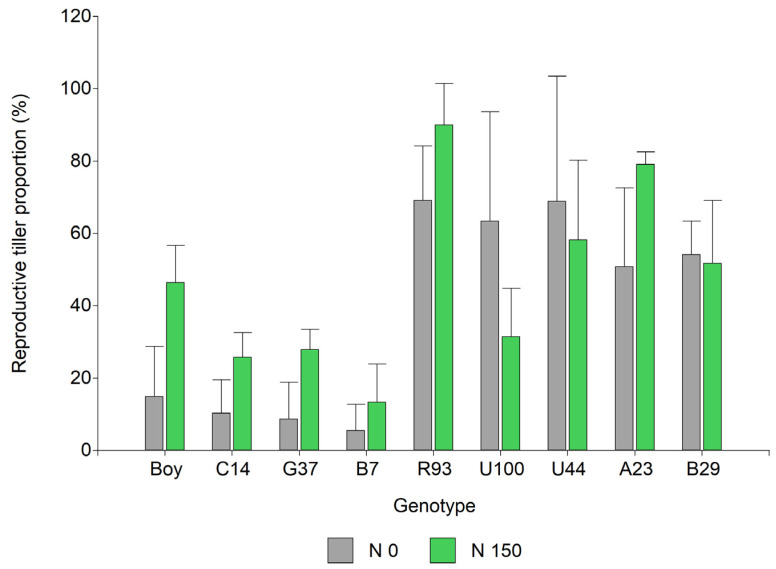
Proportion of reproductive tillers for a group of 9 *Paspalum* genotypes under two nitrogen fertilization levels, 0 (N 0) and 150 (N 150) kg N ha^−1^. Boy, C14, G37, and B7 belong to *P. notatum* (early flowering), and R93, U100, U44, A23, and B29 belong to the Plicatula Group (intermediate and late flowering). Bars represent the standard error.

The seed yield was greater for most genotypes of the intermediate- and late-flowering groups than the early-flowering group, particularly under the non-fertilized conditions. The early-flowering group was more responsive to N fertilization in comparison with the intermediate and late-flowering groups. The impact of nitrogen fertilization on *P. notatum* seed has been studied in cultivars [21,22,23] and, more recently, in novel apomictic hybrids [13,14]. A greater number of tillers under nitrogen fertilization was related to greater seed yield in *P. notatum* [13]. It was also observed that a larger fraction of tillers become reproductive under greater N availability [15]. Our results indicate that the density of reproductive tillers is the main component explaining seed yield changes in *Paspalum*. However, intermediate and late-flowering groups exhibited a greater proportion of tillers that become reproductive independently of the soil fertility (Figure 6), indicating that *P. notatum* prioritizes survival through vegetative growth under nutrient-limiting conditions.

The number of seeds per m^2^ being mainly driven by the number of reproductive tillers per m^2^ explained most changes in seed yield in *Paspalum*. These results are in agreement with previous reports related to annual and domesticated grasses like wheat [12], indicating that, in both annual and perennial grasses, the number of seed heads per m^2^ is the seed yield component that better explains changes in seed yield. However, there is a variation among perennial species in terms of the fraction of the total number of tillers that become reproductive (Figure 6).

In terms of seed quality, the intermediate- and late-flowering groups exhibited greater germination and rate of germination than the early-flowering group. This difference seems to be related to the presence of seed dormancy in *P. notatum* [24], which may not be present or reduced for genotypes of intermediate- or late-flowering groups. This is expected to have an impact on the efficiency of the relationship between seed harvested and seed needed for stand establishment. No effect of nitrogen fertilization was observed for seed set, seed germination, and the rate of germination. A few differences were observed for seed dimensions between fertilized and non-fertilized treatments, but the differences were minimal and not consistent among genotypes.

Seed shattering was markedly lower for the early-flowering group in comparison to the intermediate- and late-flowering groups. These results indicate that, while *P. notatum* does not share the typical low seed retention observed for other perennial warm-season grasses, genotypes from the Plicatula group do. These results indicate an advantage for *P. notatum* that may result in a better relation between the amount of seed produced and the amount of seed harvested. Scienza et al. [25] have also observed low seed retention for hybrids between *P. plicatulum* and *P. guenoarum*, indicating that harvest should not wait beyond 20 days after the beginning of flowering. This may be the reason why genotypes from intermediate- and late-flowering groups were far from reaching their potential seed yield, considering the seed yield components, i.e., reproductive tillers per m^2^, number of florets per tiller, and seed set. In addition, both seeds and empty spikelets are shattered in similar proportions.

In the genus *Paspalum*, early-flowering germplasm is more responsive to the addition of nitrogen in comparison to intermediate and late flowering species, responding with a more extended flowering period, greater tiller density, greater reproductive tiller density, and greater seed yield. Therefore, the addition of moderate amounts of nitrogen is necessary for seed production in *P. notatum,* as it helps maintain pastures with high tiller density and a greater proportion of tillers transitioning to reproductive stages. Conversely, if the pasture is primarily used for grazing, an extended flowering period would be undesirable, as it could lead to a lower nutritive value. Intermediate- and late-flowering species have greater seed yield potential, considering the main components of seed yield, but low seed retention directly affects the amount of seed harvested.

In conclusion, seed yield in *Paspalum* is mainly affected by the number of reproductive tillers per m^2^ and seed retention. The species differ in the amount of resources needed for the tiller’s reproductive differentiation. *P. notatum* prioritizes survival through vegetative tillers in fertility-restricted environments within a perennial life cycle. Seed retention should be considered as critical for the definition of seed harvest operations, since some species exhibited reduced levels of this trait. Functions for seed shattering over time were developed for *P. notatum* and the Plicatula group of *Paspalum*. It will be important to evaluate how the seed yield components are related to the observed variation in seed yield over years in perennial grasses. Additionally, more research is needed to determine management practices that may guarantee seed yield stability over years.

## 4. Materials and Methods

### 4.1. Plant Material

Nine genotypes of *Paspalum* were used for this research (Table 3). Four of them were intraspecific tetraploid hybrids of *Paspalum notatum*. There was an ecotype of *P. guenoarum* and three hybrids between *P. plicatulum* and *P. guenoarum*. There was also an ecotype of *P. atratum*. The rationale behind this selection was to represent the different flowering periods observed for *Paspalum* in Corrientes, Argentina. *P. notatum* usually flowers early during the growing season, *P. atratum* flowers late, and *P. guenoarum* tends to be intermediate. All of these genotypes are tetraploid and apomictic (Table 3).

### 4.2. Experimental Design

**Figure 7:**
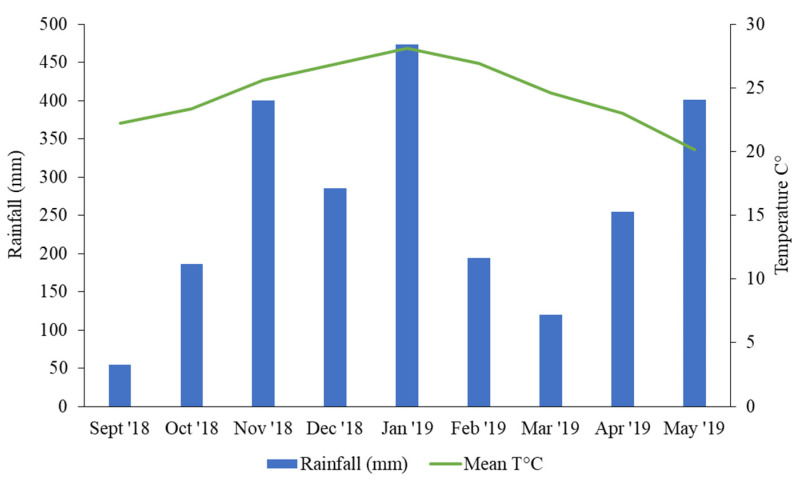
Registered rainfall and temperatures in the experimental site in Corrientes, Argentina (27°28′ S, 58°47′ W) during the evaluation period.

The experimental design was a randomized complete block design in a split-plot arrangement with three replications. The main plots (1.5 m × 13.5 m) consisted of two N levels (0 and 150 Kg N ha^−1^), and the sub-plots (1.5 m × 1.5 m) were the different genotypes (Table 1). The N treatment levels were defined based on the results reported by Adjei et al. [22]. It was expected that the application of 150 kg N ha^−1^ would have a significant impact on seed yield compared to poor sandy soil without any nutrient addition. This impact would have allowed for the investigation of critical seed yield components. The seeds were germinated in a greenhouse during the winter of 2017, and the seedlings were transplanted to seedling flats. The plants were transplanted into the field in November 2017 at the experimental field of the Universidad Nacional del Nordeste, located near the city of Corrientes, Argentina (27°28′ S, 58°47′ W). The soil type was classified as Alfic Udipsamment, characterized as a low-fertility sandy soil (93.2% sand, 4.2% slit, and 2.6% clay), well-drained, with a 0.7% land slope, and 130 cm of effective rooting zone. At the beginning of the study, the soil pH was 6.79, and Bray-I extractable P and total nitrogen were 10.4 and 500 mg Kg^−1^, respectively. The plots were cut once in the fall of 2018 during the first growing season. The evaluations were performed during the second growing season, since the plots were considered to be established. The plots were defoliated on 26 September 2018. For the fertilization treatment, the nitrogen source was urea (46-0-0). N-fertilization was conducted on 1 October 2018. The registered temperatures and rainfall during the evaluation period are presented in Figure 7.

### 4.3. Evaluated Variables

The initiation of the flowering period was determined as the number of days between the beginning of spring (26 September) and the appearance of the first inflorescence in each plot. The extent of the flowering period was calculated as the number of days between the appearance of the first and last inflorescences. During the flowering period between 27 November 2018 and 22 May 2019, the number of reproductive tillers was registered weekly using a 25 × 25 cm frame in a fixed area of each plot. Tillers were identified as reproductive based on the presence of an emerging or well-developed inflorescence. Reproductive and vegetative tiller density was determined at the end of the flowering period (when no new inflorescences were observed).

When the seeds were mature in each plot (≈20 d after flowering peak), the seed heads were manually harvested from the central 1 m^2^. The harvested material was moved to a drier provided with forced air circulation and dried at 35 °C to ~12% humidity. The seeds were manually threshed, and the seeds and empty spikelets were separated using a seed blower (Seedburo Equipment Company, Des Plaines, IL, USA). The seeds were weighed and bagged for further analysis.

Before seed harvest, a sample of 4 to 8 inflorescences was collected and dried. A series of seed yield components were estimated, including (a) the number of racemes per inflorescence, (b) the number of seeds per raceme, (c) the seed set (proportion of N° seeds in relation to the N° seeds + empty spikelets), (d) the weight of 1000 seeds, and (e) the length and width of the seed. Seed germination and the germination rate were determined following the procedure described by Maguire [27]. The germination rate was calculated based on 50 seeds in each experimental unit.

Seed shattering was evaluated using the technique developed by Tomás et al. [18]. Two inflorescences were put inside a seed trap after anthesis. Two traps were used in each plot. Shattered florets were counted every other day for 90 d; the seeds and empty spikelets were manually classified and registered. The inflorescence was removed from the trap at the end of the evaluation period, dried, and threshed. The proportion of seeds and empty spikelets that were shattered at every count was calculated, as well as the retained seed in the inflorescence at 90 days.

### 4.4. Statistical Analysis

All data generated were analyzed using the software Info-Stat 2020 [28] as a randomized complete block design in a split-plot arrangement. Means, analysis of variance (ANOVA), and means comparisons by a Tukey’s test at *p* ≤ 0.05 were calculated. The statistical significance of the interaction between the genotype and nitrogen was calculated for all traits. A principal component analysis (PCA) was performed using the mean values of each genotype across two N rates. The PCA and the biplot graphical representation were performed using the Info-Stat software.

Seed retention over time data were used to build seed-retention curves with a nonlinear log-logistic regression model:

y=D/(1+(x/I50)b

where y represents the seed retention over time x, D is the upper asymptote, I50 is the time required to 50% of the retention, and b is the slope of the line at I50. To assess the accuracy of the model, F tests for model significance, coefficient of determination (R^2^), and residual variance analysis were calculated. The models obtained were compared with the extra sum-of-squares F test using GraphPad Prism 8 (GraphPad Software, San Diego, CA, USA).

## Figures and Tables

**Table 1:** Seed yield, seed yield components, seed quality, and seed dimensions of 9 *Paspalum* genotypes cultivated on nitrogen-fertilized and non-fertilized plots. Boy, C14, G37, and B7 belong to *P. notatum* (early flowering), and R93, U100, U44, A23, and B29 belong to the Plicatula Group (intermediate and late flowering). G: Seed germination; F/i: Number flower/inflorescence.

ID	N Level	Seed Yield (g/m^2^)	N° Flowers/Raceme	N° F/i	1000 Seed Weight (g)	Tillers /m^2^	Vegetative Tillers/m^2^	Reproductive Tillers/m^2^	Seed Set (%)	G (%)	Rate G	Seed Length (mm)	Seed Width (mm)
Boy	0	8.7	93	185	3.17	837	709	192	26	2.7	0.09	3.8	2.5
	150	31.6 *	91	181	2.86	1221 *	651	571 *	25	18	0.81	3.8	2.6
C14	0	3.8	84	168	3.49	1008	901	160	34	6	0.27	3.9	2.8
	150	25 *	89	180	3.52	1371	1003	464 *	34	12.7	0.64	3.8	2.9 *
G37	0	3.8	89	177	3.36	981	885	144	32	16.7	0.66	3.7	2.8
	150	10.5	95	189	3.80	1050	763	288	32	15.3	0.56	3.9 *	2.9
B7	0	1.5	86	171	3.34	944	891	80	29	4	0.20	3.9	2.6
	150	4.5 *	91	182	3.37	1173	1013	160	29	8.7	0.33	3.9	2.6
A23	0	37.6	115	376	2.99	619	293	325	22	21.3	1.59	3.5	2.0
	150	41.5	115	372	3.13	1130 *	240	890 *	29	27.3	2.01	3.6 *	2.0
B29	0	54.4	117	423	2.96	603	272	330	27	28.7	2.07	3.1	3.1
	150	63.4 *	121	485	2.98	661	315	347	27	30.7	2.49	3.1	3.1
R93	0	47.6	113	510	2.65	587	192	347	35	39.3	2.75	3.2	2.0
	150	48.3	117	607	2.53	720	101	736 *	23	30.3	2.35	3.2	2.0
U100	0	5.7	74	486	3.44	331	123	208	12	47.3	4.13	3.5	2.1 *
	150	13.1	84	549	3.36	848 *	586	261	19	45.3	4.06	3.4	2.0
U44	0	54.9	77	438	3.15	544	245	299	36	44	3.72	2.0	3.0
	150	68.4	74	458	3.20	880	432	448	42	39.3	3.27	2.0	3.0
MSD ^†^	0	30.1	13	76	0.65	577	524	299	22	20	1.4	0.14	0.12
	150	24.4	12	88	0.60	714	524	393	12	28	2.1	0.12	0.11

* significant difference between nitrogen levels in genotypes by Tukey test (*p* ≤ 0.05). ^†^ MSD: minimum significant differences for comparisons between genotypes into a given nitrogen level by Tukey test (*p* ≤ 0.05).

**Table 2:** Seed retention for 9 *Paspalum* genotypes cultivated on nitrogen-fertilized and non-fertilized plots. Boy, C14, G37, and B7 belong to *P. notatum* (early flowering), and R93, U100, U44, A23, and B29 belong to the Plicatula Group (intermediate and late flowering). E/S: empty spikelets; F/S: full seeds.

ID	N Level	E/S Retention 20 Days (%)	F/S Retention 20 Days (%)	E/S Retention 40 Days (%)	F/S Retention 40 Days (%)	E/S Retention 60 Days (%)	F/S Retention 60 Days (%)	E/S Retention 90 Days (%)	F/S Retention 90 Days (%)
Boy	0	98 a^†^	100 a	81 abc	100 a	97 a	98 a	97 a	98 a
	150	99 a	100 a	97 ab	99 a	99 a	99 a	99 a	99 a
C14	0	100 a	100 a	100 a	100 a	96 a	98 a	96 a	98 a
	150	100 a	100 a	98 a	100 a	93 a	97 a	93 a	97 a
G37	0	100 a	100 a	99 a	98 a	95 a	96 a	95 a	96 a
	150	100 a	100 a	99 a	100 a	97 a	99 a	98 a	99 a
B7	0	100 a	100 a	99 a	100 a	94 a	98 a	94 a	96 a
	150	100 a	100 a	100 a	100 a	100 a	99 a	99 a	99 a
A23	0	96 ab	86 a	72 abc	73 ab	50 bcd	62 b	13 bc	12 c
	150	86 b	77 a	49 c	55 b	28 cde	23 cde	19 cd	13 c
B29	0	93 ab	89 a	56 abc	40 bcd	31 cde	26 cd	8 de	6 d
	150	95 ab	80 a	75 abc	48 bcd	59 bc	40 c	32 b	19 b
R93	0	95 ab	78 a	60 abc	17 d	42 bcde	10 de	5 cd	3 de
	150	92 ab	82 a	59 abc	35 cd	35 cde	28 cd	4 cd	2 de
U100	0	95 ab	85 a	54 abc	36 cd	12 e	5 e	2 d	1 de
	150	92 ab	81 a	53 bc	30 cd	16 de	11 de	3 d	2 de
U44	0	96 ab	96 a	67 abc	33 cd	31 cde	25 cde	0 d	0 e
	150	97 ab	88 a	81 abc	31 cd	21 de	18 de	1 d	1 de

^†^ Means followed by different lowercase letters are different by LSD’s test (*p* < 0.05) within a given column.

**Table 3:** List of *Paspalum* genotypes used for this research. All of them are tetraploid and apomictic.

ID	Origen	Flowering Period
Boy	*P. notatum* cv Boyero UNNE [26]	Early
C14	Intraspecific hybrid of *P. notatum* [9]	
G37	Intraspecific hybrid of *P. notatum* [9]	
B7	Intraspecific hybrid of *P. notatum* [9]	
B29	Hybrid between *P. plicatulum* and *P. genoarum* Baio [10]	Intermediate
R93	Hybrid between *P. plicatulum* and *P. genoarum* Rojas GR19 [10]	
A23	Hybrid between *P. plicatulum* and *P. genoarum* Azulao [10]	
U100	*P. guenoarum* ecotype	
U44	*P. atratum* ecotype	Late

## Data Availability

Data is contained within the article.
